# Robust dynamic balance of AP-1 transcription factors in a neuronal gene regulatory network

**DOI:** 10.1186/1752-0509-4-171

**Published:** 2010-12-17

**Authors:** Gregory M Miller, Babatunde A Ogunnaike, James S Schwaber, Rajanikanth Vadigepalli

**Affiliations:** 1 Daniel Baugh Institute for Functional Genomics and Computational Biology,Department of Pathology, Anatomy and Cell Biology, Thomas Jefferson University Philadelphia, PA, 19107, USA; 2 Department of Chemical Engineering, University of Delaware Newark, DE, 19716, USA

## Abstract

**Background:**

The octapeptide Angiotensin II is a key hormone that acts via its receptor AT1R in the brainstem to modulate the blood pressure control circuits and thus plays a central role in the cardiac and respiratory homeostasis. This modulation occurs via activation of a complex network of signaling proteins and transcription factors, leading to changes in levels of key genes and proteins. AT1R initiated activity in the nucleus tractus solitarius (NTS), which regulates blood pressure, has been the subject of extensive molecular analysis. But the adaptive network interactions in the NTS response to AT1R, plausibly related to the development of hypertension, are not understood.

**Results:**

We developed and analyzed a mathematical model of AT1R-activated signaling kinases and a downstream gene regulatory network, with structural basis in our transcriptomic data analysis and literature. To our knowledge, our report presents the first computational model of this key regulatory network. Our simulations and analysis reveal a dynamic balance among distinct dimers of the AP-1 family of transcription factors. We investigated the robustness of this behavior to simultaneous perturbations in the network parameters using a novel multivariate approach that integrates global sensitivity analysis with decision-tree methods. Our analysis implicates a subset of Fos and Jun dependent mechanisms, with dynamic sensitivities shifting from Fos-regulating kinase (FRK)-mediated processes to those downstream of c-Jun N-terminal kinase (JNK). Decision-tree analysis indicated that while there may be a large combinatorial functional space feasible for neuronal states and parameters, the network behavior is constrained to a small set of AP-1 response profiles. Many of the paths through the combinatorial parameter space lead to a dynamic balance of AP-1 dimer forms, yielding a robust AP-1 response counteracting the biological variability.

**Conclusions:**

Based on the simulation and analysis results, we demonstrate that a dynamic balance among distinct dimers of the AP-1 family of transcription factors underlies the robust activation of neuronal gene expression in the NTS response to AT1R activation. Such a differential sensitivity to limited set of mechanisms is likely to underlie the stable homeostatic physiological response.

## Background

The present study aims to understand molecular neuronal processes relevant to hypertension, involving angiotensin II (Ang II) type 1 receptor (AT1R) signaling as it regulates production of Tyrosine hydroxylase (TH). The nucleus tractus solitarius (NTS), located in the brainstem, is critically involved in the regulation of blood pressure [1]. AT1R signaling within the NTS has been associated with disturbances of autonomic homeostasis, including essential hypertension [2-8]. The most effective current pharmaceutical agents treating hypertension target AT1R. Norepinephrine production and release, involving the A2 catecholamine neuronal population in the NTS, is also involved in NTS regulation of blood pressure and the development of hypertension [9-11]. TH is the rate-limiting enzyme for norepinephrine transmitter production by the A2 neurons, and its regulation is a plausible mechanism for the effects of AT1R on NTS function in determining the level of blood pressure [12-14].

Ang II binding to AT1R in the brain contributes to homeostatic processes through activation of a cascade of signaling proteins that regulate a complex network of transcription factors (TFs) and their target genes via multiple feedback loops [13,15-19]. More recently, evidence for this complexity of response was obtained by analyzing the transcriptomic response of NTS neurons to hypertension [20] using our PAINT bioinformatics approach [21-23]. We used these NTS and hypertension-specific network hypotheses along with the broad literature on AT1R effects on the brainstem (detailed below) towards developing a gene regulatory network structure underlying AT1R-driven molecular processes in the NTS adapting to hypertension. This experimentally motivated regulatory network structure provides a qualitative description of connectivity and potential control points.

In order to provide understanding of the dynamics of AT1R-activated molecular processes we now take a modeling approach that integrates experimental data with model predicted dynamics of critical network components in order to gain insights into the network behavior. We would like to understand which of the dynamic profiles of the signaling kinases, downstream of AT1R activation, are significant control factors over time as they propagate through the regulatory network and contribute to the downstream activation of the TF AP-1. Of key interest is identifying how the potential controlling factors interact with each other to shape the downstream TF dynamics. Insights into these issues will significantly aid our understanding of how the signaling kinase response to AT1R activation leads to activation of downstream TFs and their target genes.

The complexity of the network and network dynamics, and of the required perturbations, yields a large combinatorial functional space. We follow an integrated approach that includes dynamic modeling and a novel combination of global sensitivity analysis and decision tree construction methods towards identifying key network interactions and their dynamical relationships. By this approach, we are able to systematically investigate the implications of many potential perturbations on AP-1 activity dynamics. The decision tree methods are not scalable to the large number of parameters in the present model. Hence, we follow a sequential approach to first identify key parameters through global sensitivity analysis and then identify the conditional relationships among these parameters using the decision tree methods. The decision trees characterize the potential for biological variability, as different neurons may take a different path, yielding distinct classes of AP-1 activity dynamical responses.

Our analysis reveals a complex higher-order interaction between key network parameters. We interpret these in the context of the network structure to derive insights into how heterogeneity of neuronal state and parameters can affect the predicted dynamic balance of distinct AP-1 dimer activation. Our analysis significantly reduces the dimensionality of the set of potential key network components and interactions to develop a focused set of hypotheses for experimental validation. Of note, we find that the dynamics of the sensitivities in the network is synchronized with the dynamic balance of distinct AP-1 dimer forms.

The remainder of the manuscript is organized as follows: We first present the details of the model formulation including experimental results motivating the model structure, assumptions made in model development, and fitting model parameters to the available experimental data. We then present the model simulation results including validation using kinase inhibitor experiments. Next section presents results from the global sensitivity and decision tree analyses. Each of these sections is contextually organized into subsections that focus on specific model aspects and simulation and analysis results. All the technical methods are detailed in the Methods section. Model equations and parameter values are detailed in the Additional file 1.

## Results and Discussion

### Model Formulation: AT1R activated gene regulatory network

In order to gain quantitative insights into the regulatory mechanisms underlying the dynamics of TH gene expression, we developed a computational model of the gene regulatory network activated by Ang II through its receptor AT1R in the NTS. Our previous high-throughput transcriptomic study showed evidence for regulation of AT1R initiated signaling in the NTS in response to an acute hypertensive stimulus [20]. Pathway analysis of this data identified differential activity of PKCα, extracellular signal-regulated kinase (ERK), and c-Jun N-terminal kinase (JNK) pathways as involved in the NTS response. Promoter analysis of these gene expression profiles using our PAINT bioinformatics software [21,23] indicated AP-1 as a key regulator in this adaptive process [20]. These findings are inline with the extensive experimental literature on the role of AT1R and downstream signaling pathways in NTS regulation of blood pressure (see reviews [8,18,19,24]).

**Figure 1:**
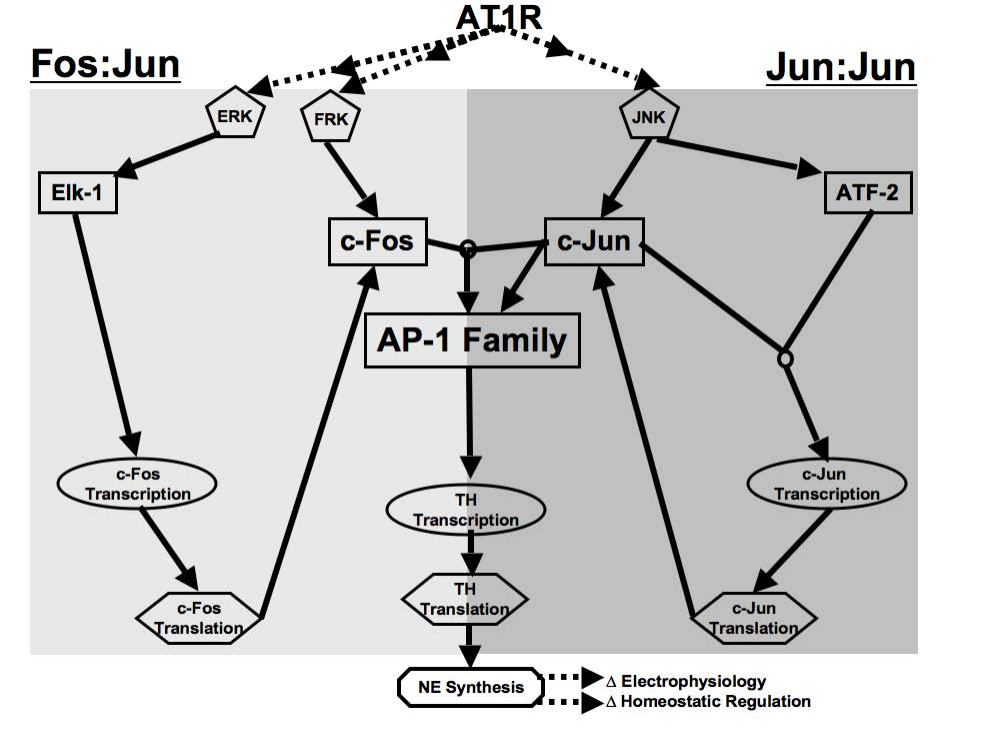
**Schematic representation of the AT1R-modulated gene regulatory network model**. Ang II binding to AT1R leads to activation of signaling kinases (ERK, FRK, and JNK), which regulate the AP-1 family of transcription factors through fast (protein phosphorylation) and slow (protein synthesis) cellular processes. Activated signaling kinases phosphorylate downstream transcription factors (Elk-1, c-Fos, c-Jun, ATF-2) leading to their subsequent DNA-binding activity. Active AP-1 family of transcription factors considered here are the heterodimers of dual phosphorylated c-Fos and c-Jun or homodimers of dual phosphorylated c-Jun. These AP-1 forms induce transcription of Tyrosine hydroxylase (TH), a critical regulator of neuronal function. Reactions leading to activation of ppc-Fos:ppc-Jun are highlighted in light grey, and reactions leading to activation of ppc-Jun:ppc-Jun in dark grey.

Stimulation of AT1R leads to increased activity of signaling kinases FRK, ERK, and JNK [25,26], which are transcriptional and post-translational regulators of the AP-1 family of TFs [27]. FRK and JNK alter the phosphorylation state of AP-1 subunits c-Fos and c-Jun, respectively [27]. JNK plays an additional role in AP-1 activation by phosphorylating ATF-2, a subunit of the c-Jun TF c-Jun:ATF-2 [27]. ERK affects AP-1 transcriptionally by phosphorylating Elk-1, a TF regulating c-Fos [27]. AP-1 phosphorylation enables its DNA-binding to its regulatory site in the TH gene promoter, thus inducing TH transcription [24,27]. A schematic of this network is depicted in Figure 1 and is the initial structural basis for our model formulation and computational analysis.

**Figure 2:**
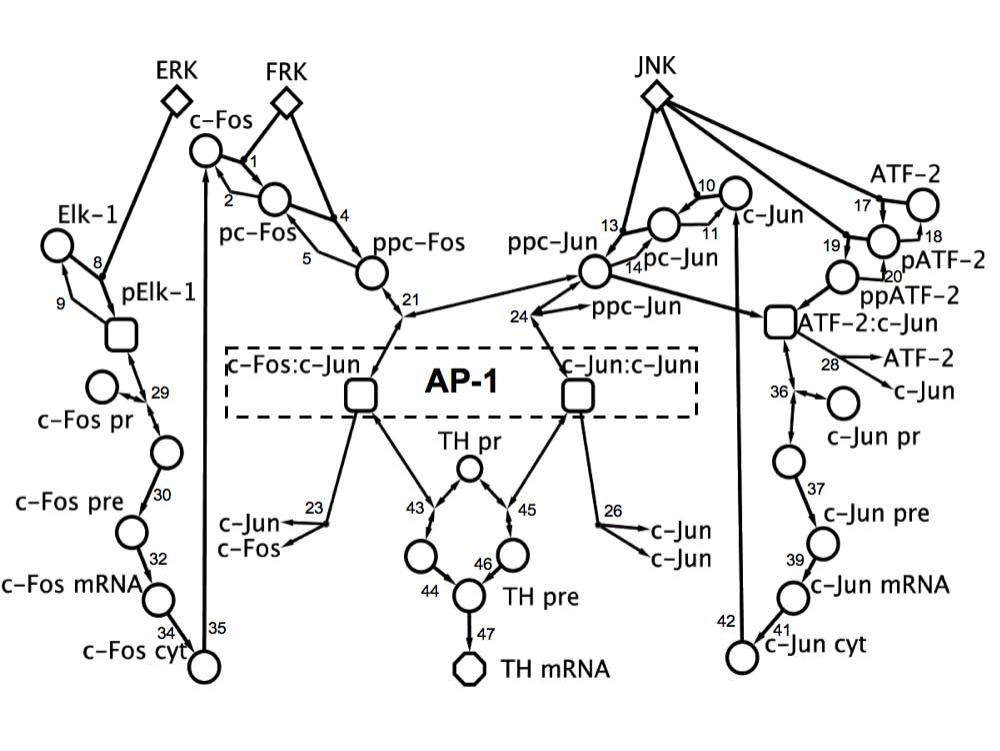
**AT1R gene regulatory network model wiring diagram**. Nodes represent model species and edges depict chemical reactions. The reaction numbers correspond to those in Additional file 1, Table S1. Kinases have been represented by diamonds, active transcription factors by rounded rectangles, TH mRNA by a hexagon, and the remaining species shown as circles. Total AP-1 is indicated by the box comprising ppc-Fos:ppc-Jun and ppc-Jun:ppc-Jun. Degradation reactions and protein:DNA complexes have been omitted from this diagram. These correspond to reactions 3, 6, 7, 12, 15, 16, 22, 25, 33, 40 and 48. A full description of model reactions can be found in Additional file 1, Tables S1 and S2.

Our model, which we refer to as AT1RGRN (AT1R Gene Regulatory Network) considers 32 species, 29 of which are represented by ordinary differential equations, and the dynamics of the remaining three species (signaling kinases ERK, FRK and JNK) are modeled using experimental data time series from the literature. AT1RGRN contains 77 parameters including non-zero initial conditions of several model species, and is compartmental, simulating chemical reactions occurring in nuclear and cytosolic fractions. A detailed view of the reaction scheme is presented in Figure 2. Notable features of our model include activation of immediate early TFs by signaling kinases, combinatorial interactions among the TFs to yield active dimer forms, and a subset of these TFs influencing their own dynamic activity via regulation of corresponding genes. AT1RGRN contains two key modules: Fos and Jun dependent, and Fos independent but Jun dependent (Figure 1). The detailed kinetic reactions underlying this modular structure are shown in Figure 2, with the notable characteristic being the Fos and Jun hetero-dimerization and Jun homo-dimerization to yield two distinct forms of active AP-1. A summary of key features of the model is given in the following subsections. The complete mathematical formulation of the model is given in the Additional file 1, Figures S1 and S2.

#### Linking AT1R activation to signaling kinases

Potential factors connecting AT1R activation to the nucleus and therefore serving as downstream transmitters of the AT1R binding signal are the signaling kinases FRK, ERK, and JNK [19,26,28]. Rather than explicitly describing the complex mechanisms linking receptor activation to signaling kinases, we make an approximation using available experimental data measuring the dynamics of kinase activities reported in response to identical experimental conditions using cultured neurons. Our approach to limit the complexity to the downstream gene regulatory network dynamics makes the present study tractable. In addition, the time scales of interest in the present study are 0-60 min post AT1R activation, allowing us to neglect potential feedback effects of gene regulation on AT1R signaling pathways. The experimental data on FRK, JNK and ERK time series was obtained from [25,26]. These profiles are based on neuronal response to AT1R activation by 100 nM Ang II for at least 60 min. All the kinases show a unimodal response. Kinase activity data normalized to their maximal levels is shown in Additional file 1, Figure S1.

We assume that the reported kinase activities correspond to that in the nuclear compartment. We initially assume that the nuclear concentrations of FRK, ERK, and JNK are of similar order of magnitude. Following this assumption, we performed initial model simulations with equal maximum concentration of input kinases. We relax this assumption in our global sensitivity analysis by considering these maximal concentrations as parameters for independent perturbation. As detailed below, our model analysis suggests these parameters play a significant role in shaping the AT1RGRN output over time.

#### Capturing complex network processes using simplifying assumptions

The gene regulatory network modulated by AT1R involves a potentially large number of molecular players, and we model the present understanding of their reaction kinetics based on simplifying assumptions similar to those used in typical signaling model development [29,31]. These allow the model parameters estimation based on the available experimental data, as follows:

i) Phosphorylation and dephosphorylation reactions were approximated by Michaelis-Menten kinetics.

ii) Phosphatase concentrations were not modeled explicitly, and instead their availability was assumed to be constant during the time window of interest, i.e., 60 minutes of Ang II stimulus.

iii) Dissociation and dephosphorylation of phosphorylated dimers were approximated by a single reaction step with Michaelis-Menten kinetics.

iv) The complex molecular processes connecting transcription factor DNA-binding to increased protein synthesis were represented by model reactions using elementary mass-action and first-order kinetics. Active TFs bind their target gene promoters to form TF:promoter complexes, modeled according to mass-action kinetics.

v) Precursor mRNA (pre-mRNA) was assumed to be transcribed from TF:promoter complexes with first-order kinetics.

vi) pre-mRNA processing followed by translocation to the cytosol was modeled by a single first-order kinetic process transforming nuclear pre-mRNA to cytoplasmic mRNA.

vii) Proteins were assumed to be translated from mRNA through a first-order kinetic process.

#### Total AP-1 dynamic activity as the network output of interest

The dynamic functional role of various TFs involved in Ang II mediated neuronal adaptation are not well understood. In the present work, we focus on the AP-1 family of TFs and particular members of this family are regulated in an interacting network, and the consequences on downstream target gene expression of TH. We consider two distinct AP-1 dimers: the heterodimer ppc-Fos:ppc-Jun, and the homodimer ppc-Jun:ppc-Jun (Note: the prefix 'pp' indicates the dual phosphorylation state of the corresponding proteins c-Fos and c-Jun).

Recent experimental work characterized the AT1R-induced activation of AP-1 using a technique that measured a net DNA-binding activity of AP-1 proteins using electrophoretic mobility shift assays that measure binding to labeled double stranded DNA oligo containing a canonical AP-1 binding site sequence (Fleegal and Sumners, 2003). As this experimental data is unable to differentiate between ppc-Fos:ppc-Jun and ppc-Jun:ppc-Jun, in our model fitting and subsequent analysis we define an aggregate "Total AP-1" as a sum of the activities of these two AP-1 forms.

#### Model Fitting

It should be noted that identifying exact parameter values in this model fit is not critical as we investigate the model-predicted dynamical mechanisms and interactions through a global sensitivity analysis that is based on simultaneous perturbations in all of the model parameters. Initial model parameters were adjusted to fit the model predicted Total AP-1 activity dynamics to the experimentally measured response in the literature, as described in the Methods section. In brief, we began with base parameter values from similar reactions described in previous modeling studies that are based on neuronal and other cell types [29,30,32]. Parameters were then varied simultaneously to fit AT1RGRN to the experimentally measured data from brainstem neuronal cultures: pElk-1 DNA binding [33], c-Fos mRNA [34], AP-1 DNA binding [35], and TH mRNA [28]. A model fit to these experimental data is shown in Additional file 1, Figure S1. The corresponding model parameters are given in Additional file 1, Table S3.

### Model Simulations: Ang II Activates AP-1 Through a Dynamic Balance of TF Dimers

**Figure 3:**
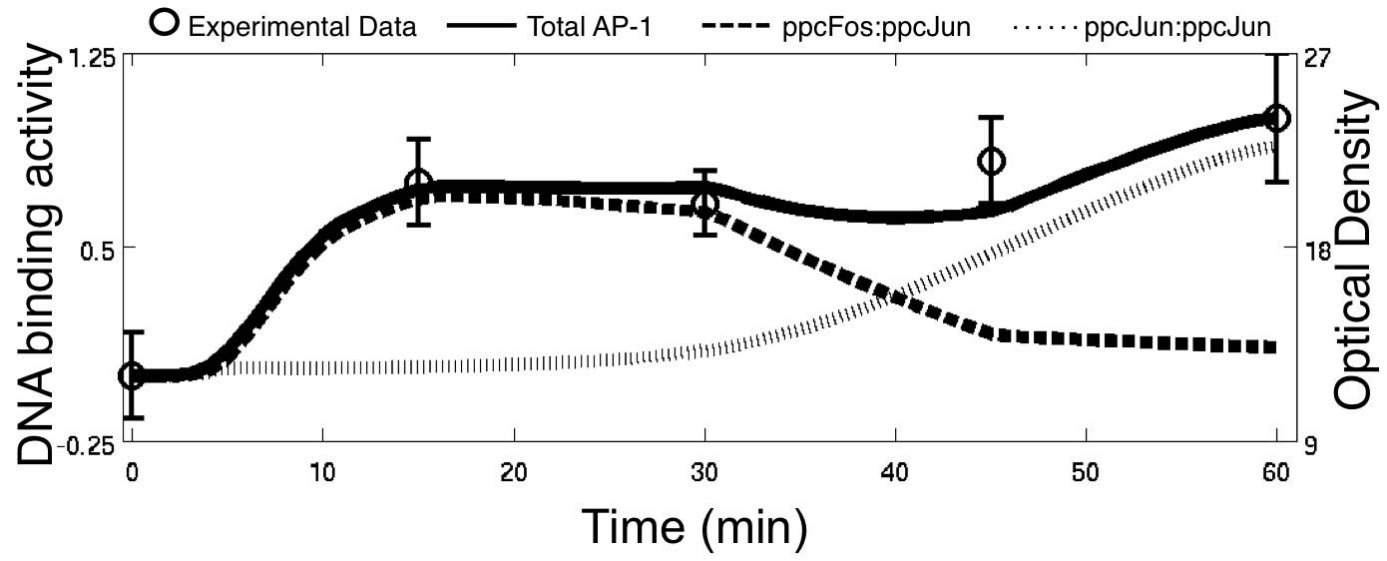
**AP-1 activation in response to Ang II treatment**. Simulated normalized Total AP-1 activity (solid line) corresponds to the sum of ppc-Fos:ppc-Jun (heavy dashed line) and ppc-Jun:ppc-Jun (light dashed line), and can be compared to experimentally measured AP-1 DNA binding activity (circles with error bars) in the literature [35]. Experimental data is shown according to the reported numerical values of Optical Density measurements (right axis). Model-predicted AP-1 levels have been normalized according to the normalization procedures described in the Methods section.

The AT1GRN predicted AP-1 activity dynamics in response to a 100 nM Ang II stimulus is shown in Figure 3. The total AP-1 activity response is consistent with experimental observations [35]. These model simulations reveal that the activated AP-1 activity response arises from a dynamic balance of constituent AP-1 dimer forms. Our model predicts that AP-1 is activated initially in the form of ppc-Fos:ppc-Jun, which achieves a maximum activity at approximately 15 minutes. Subsequently, the response begins to shift away from Fos-Jun heterodimer (ppc-Fos:ppc-Jun) to an increased contribution from the Jun homodimer (ppc-Jun:ppc-Jun). This dynamic shift continues even after 40 minutes, when the AP-1 response is dominated by ppc-Jun:ppc-Jun. Interestingly, our model simulations predict that the AP-1 response to arises from an equal contribution of ppc-Fos:ppc-Jun and ppc-Jun:ppc-Jun at approximately 40 minutes of AngII stimulus.

The observed inverse correlation between ppc-Fos:ppc-Jun and ppc-Jun:ppc-Jun based mechanisms arises from two aspects of the regulatory network: (1) the affinity for AP-1 formation favors ppc-Fos:ppc-Jun heterodimers over ppc-Jun:ppc-Jun homodimer forms, as supported by experimental findings (e.g., [15,16,32]); and (2) FRK activation decreases earlier than that of JNK, leading to reduced availability of active ppc-Fos for binding to ppc-Jun. Active JNK leads to continued activation of higher concentrations of c-Jun built up in this period that overcomes the affinity barrier to form active AP-1 homodimers. In the following sections, we visualize and interpret these processes in the context of sensitivity of the AP-1 activity and the predicted dynamic balance to simultaneous perturbations in model parameters. We use a global sensitivity analysis followed by decision tree methods to test the hypothesis that persistent AP-1 activity through the predicted dynamic balance of constituent dimer forms is robust to most parameter changes in the network.

### Model Validation through Pathway Inhibitor Effects on AT1R-induced AP-1 Activation

We explored the role of the kinases FRK and JNK on AP-1 activity by simulating the effect of Ang II treatment coupled with pathway inhibitors to prevent kinase activation above basal levels. The motivating concern is whether the model-predicted dynamic balance is of relevance beyond the nominal system behavior and whether it can capture information other than what was used to fit the nominal model. Hence, we use the experimental data on AP-1 activity downstream of AT1R activation in the presence of pathway inhibitors as a validation data set [35]. The kinase inhibitor treatment was simulated by setting the corresponding kinase activity profile to the basal levels. Our simulations reveal that the effect of inhibiting input kinases on AP-1 output is strongly dependent on the choice of perturbed pathway and the time point post Ang II stimulus. These results detailed below provide insights in interpreting experimental observations that otherwise yielded multiple competing hypotheses on whether c-Fos plays a role in AP-1 activated downstream of AT1R.

#### Inhibiting FRK eliminates early Total AP-1 activation, but preserves later activation

**Figure 4:**
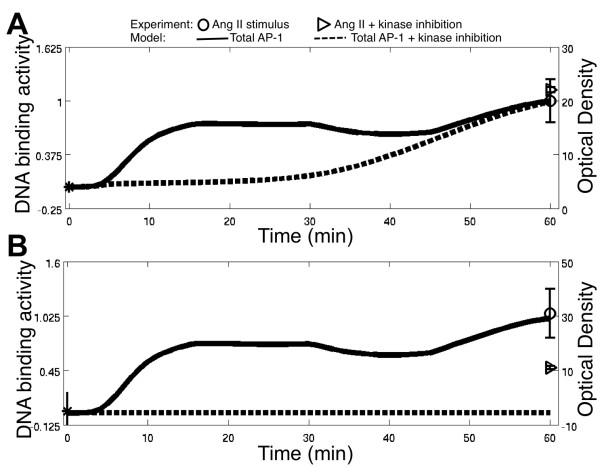
**Effect of kinase inhibitors on Total AP-1 DNA binding activity dynamics**. Ang II + kinase inhibitor treatment was simulated by setting the corresponding kinase activity profile to the basal levels present in the absence of Ang II treatment. Experimental data from pathway inhibitor studies were taken from [35] and plotted as the originally reported Optical Density measures (right axis). Experimental data is based on pretreatment of the neuronal cultures (A) with 10 uM chelerythrine chloride, an FRK inhibitor, (B) or with JNK inhibitor II, for 30 minutes prior to Ang II stimulus. **(A)** Inhibition of FRK. **(B)** Inhibition of JNK. Symbols: The control unstimulated AP-1 activity at time zero (asterisk), 60 min AP-1 activity in response to 100 nM Ang II stimulus (circle with error bars), 60 min AP-1 activity in response to 100 nM Ang II stimulus in neurons pretreated with a kinase inhibitor (triangle), simulated normalized AP-1 activity in response to Ang II (solid line) and Ang II + kinase inhibition (heavy dashed).

In our model simulations, inhibiting FRK signaling prevents the phosphorylation of c-Fos, thus causing a shift in the AP-1 dimer composition to be exclusively ppc-Jun:ppc-Jun. The question we chose to answer is whether activation of Jun homodimer alone can produce AP-1 activity that is consistent with experimental observations. As shown in Figure 4A, model simulations reveal that the activation of AT1R with FRK inhibition eliminates the AP-1 response during the first 20 minutes, while restoring the AP-1 response to the uninhibited levels at late time points. This prediction is confirmed by experimental data showing an insignificant difference between the AP-1 activity in neurons treated with Ang II and FRK inhibitors and the AP-1 activity in neurons treated with Ang II alone after 60 minutes [35]. The dynamic balance of AP-1dimers predicted by AT1RGRN simulation support this result that suggests the 60 minute AP-1 response is insensitive to perturbations in FRK due to the shift toward ppc-Jun:ppc-Jun by this time. We explore this issue further in the global sensitivity analysis and decision tree approach detailed below.

#### Inhibiting JNK eliminates Total AP-1 activation throughout AT1R stimulation

We followed a similar approach as above to investigate the contribution of JNK signaling on the activation of AP-1. As suggested by the network connectivity of the AT1GRN, inhibition of JNK signaling prevents the phosphorylation of c-Jun and ATF-2, leading to a complete elimination of ppc-Fos:ppc-Jun and ppc-Jun:ppc-Jun throughout Ang II treatment (Figure 4B). This model prediction is confirmed by experimental data showing a significant decrease in AP-1 activity (to near nominal levels, with no statistically significant difference) in neurons treated with Ang II and JNK inhibitors [35].

These validation results indicate that the dynamic balance predicted by the model is not an artifact of model fitting to the nominal case, but also underlies system response to novel perturbations and consistent with experimental observations in such cases. In particular, our model simulations reveal that the experimentally observed AP-1 sensitivities to pathway inhibitors should be interpreted with caution as these effects are dynamic and yield contrasting results based on the time point of interest.

### Network Analysis: Sensitivity of AP-1 Activity is Dynamic and Related to the Underlying Balance of c-Fos and c-Jun Dependent Mechanisms

When investigating the impact of specific reaction mechanisms on AP-1 activity, a common approach is to employ local sensitivity analysis to consider the effects of perturbing one reaction in isolation while setting all other reaction mechanisms to their nominal values [31,36,37]. However, these approaches are not well suited to address how multiple perturbations affect network output, as well as how robust network dynamics arise from heterogeneity in which several parameters are altered simultaneously across cells, tissues, and animals. In this study, we describe a multivariate approach using global sensitivity and decision-tree analysis to investigate how simultaneous perturbations in multiple reaction mechanisms affect AP-1 activity dynamics.

#### Variance-based Global Sensitivity Analysis

Understanding how network perturbations, taken as deviations in reaction rate parameters and initial conditions from their nominal values, affect AP-1 activity is difficult because of the presence of nonlinear, interacting reaction mechanisms acting at multiple time scales. Here we apply a variance-based global sensitivity analysis approach originally developed by [38] and numerically implemented by [39] to understand the effect of individual network reactions and their interactions on AP-1 activity dynamics.

The global sensitivity analysis approach is detailed in the Methods section. Briefly, we compute two indices based on simulated time series data corresponding to a large number of parameter perturbations: The first order sensitivity index $S_{1}^{i}$ captures the fractional variance of AP-1 activity that is directly attributable to changes in the parameter *i*, independent of changes in other parameters. The total effect sensitivity index $S_{T}^{i}$ represents the fractional variance of AP-1 activity attributable to changes in the parameter *i*, and all of its interactions with changes in other parameters. The difference between the two indices provides an estimate of higher order effects of parameter perturbations in which particular combinations of parameters significantly affect AP-1 activity.

#### Differential dynamic sensitivity of Total AP-1 activity

**Figure 5:**
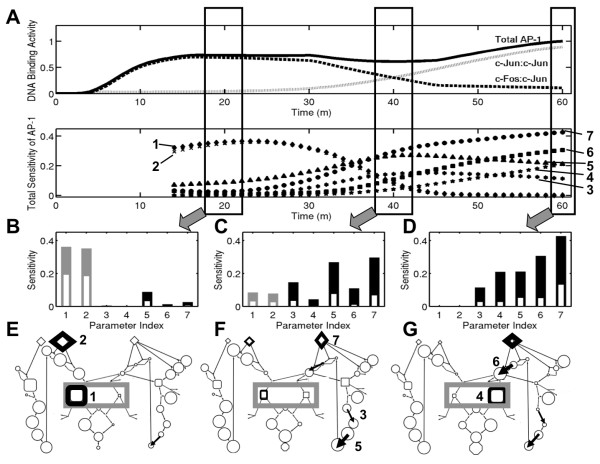
**Sensitivity of AP-1 to perturbations in network parameters**. **(A)**Upper Panel: Simulated response of Total AP-1 DNA binding activity as well as its constituent dimers to Ang II stimulus. Note that these profiles are repeated from Figure 3 to facilitate direct interpretation of the AP-1 activity time series in the context of sensitivity analysis. The composition of Total AP-1 activity has been highlighted by the rectangles at three time points of interest. Lower Panel: Dynamic profiles of total-effects sensitivity indices of Total AP-1 for the top seven most sensitive model parameters (1: ppc-Fos:ppc-Jun dephosphorylation, 2: FRK dynamics, 3: c-Jun pre-mRNA synthesis, 4: ppc-Jun:ppc-Jun dephosphorylation, 5: c-Jun translation, 6: pc-Jun phosphorylation, 7: JNK dynamics). **(B-D)** Contribution of first-order effects (white inset) to the AP-1 activity total effects sensitivities calculated at (B) 20, (C) 40, and (D) 60 minutes after Ang II treatment. Gray and black bars correspond to Fos:Jun and Jun:Jun arms of the network, respectively. Parameter index matches the ordering in (A). Bar coloring corresponds to mechanisms relating to activation of ppc-Fos:ppc-Jun (light grey) or ppc-Jun:ppc-Jun (black) as shown in Figure 2. **(E-G)** DyNSIM representation of global sensitivity analysis results in network context at (E) 20, (F) 40, and (G) 60 minutes after Ang II treatment. Total AP-1 activity is indicated in the purple rectangle, while line thickness indicates sensitivity of Total AP-1 activity to the reaction mechanisms index of (A). A detailed description of network nodes is presented in Figure 2. Sensitivity analysis of Total AP-1 was performed during 15-60 minutes of Ang II treatment when the variance of simulated Total AP-1 activity levels was greater than 1% of the maximum variance to avoid numerical oddities affecting the sensitivity calculations.

Global sensitivity analysis reveals AP-1 activity to be highly sensitive to a small set of AT1RGRN parameters. The dynamics of *S*_*T *_of AP-1 for the most sensitive model parameters are presented in Figure 5A, while *S*_*T *_for all parameters are given in Additional file 1, Figure S3. Note that the results prior to the 14 min time point are not reliable for interpretation of sensitivities as the variance estimates are too low (Additional file 1, Figure S2). As shown in Figure 5, global sensitivity analysis suggests that the dynamically switching activation of AP-1 TF dimers is achieved by shifting sensitivity and balance of network interactions. AP-1 is predicted to be activated as ppc-Fos:ppc-Jun at early times, when global sensitivity analysis finds AP-1 to be most sensitive to the development and maintenace of ppc-Fos:ppc-Jun, before shifting to be activated as ppc-Jun:ppc-Jun at later times, when the most sensitive reactions are related to the development and maintenance of ppc-Jun:ppc-Jun.

Additionally, the dynamics of several *S*_*T *_profiles shown in Figure 5A resemble closely the predicted dynamics of activated AP-1 TF dimers. The dynamics of *S*_*T *_of AP-1 to ppc-Fos:ppc-Jun dephosphorylation and FRK dynamics, which are reaction mechanisms related to the activation and maintenance of ppc-Fos:ppc-Jun, correlate well with the ppc-Fos:ppc-Jun activation predicted with high sensitivity at early times that becomes lower as the AT1RGRN simulation proceeds. Similarly, the dynamics of *S*_*T *_of AP-1 to ppc-Jun:ppc-Jun dephosphorylation pc-Jun phosphorylation, and JNK dynamics, which are reaction mechanisms related to the activation and maintenance of ppc-Jun:ppc-Jun, resemble closely the dynamics of ppc-Jun:ppc-Jun activation.

In contrast to the reaction mechanisms described above, the dynamics of *S*_*T *_of AP-1 to c-Jun translation and c-Jun pre-mRNA synthesis do not resemble the dynamics of either ppc-Fos:ppc-Jun or ppc-Jun:ppc-Jun. Instead, AP-1 is insensitive to these reaction mechanisms at early times, while becoming increasingly sensitive at later times until reaching a maximum sensitivity around 40 minutes followed by a decrease in sensitivity during the late AP-1 response. Interestingly, the time when AP-1 is most sensitive to these mechanisms is almost exactly the time in which AP-1 is predicted to be composed of equal parts ppc-Fos:ppc-Jun and ppc-Jun:ppc-Jun TF dimers. This transient sensitivity suggests c-Jun synthesis is rate limiting in initial ppc-Jun:ppc-Jun formation, before becoming less important towards the end of AT1RGRN simulation at which time c-Jun has been sufficiently produced to enable ppc-Jun:ppc-Jun formation.

Notably, the parameters corresponding to ERK activity and immediate downstream processes were not among the key set identified by the multivariate analysis (Figure 5A and Additional file 1, Figure S3). The parameters of FRK and JNK mediated processes more directly affect the active protein levels of Fos and Jun, whereas ERK affects c-Fos transcription indirectly affecting the corresponding protein levels. This may explain why the contribution of ERK-based processes to the variance in AP-1 DNA binding activity is relatively lower than that of FRK and JNK mediated processes.

#### First-order influences dominate the early AP-1 response, while interactions affect intermediate and late AP-1 activity dynamics

In the following, we focus our attention on the AP-1 response at three critical times after AT1R activation: early (20 minute), intermediate (40 minute), and late (60 minute). These time points were selected because they represent three distinct states of AP-1 activation: AP-1 activated as ppc-Fos:ppc-Jun (early); AP-1 activated as equal parts ppc-Fos:ppc-Jun and ppc-Jun:ppc-Jun (intermediate); and AP-1 activated as ppc-Jun:ppc-Jun (late). In addition, we classify reaction mechanisms as being either related to c-Fos or c-Jun based on their location on the AT1RGRN network diagram shown in Figure 1. The *S*_1 _and *S*_*T *_of the most sensitive AT1RGRN mechanisms on AP-1, as identified in Figure 5A, at these time points is presented in Figures 5B, C, and 5D.

By considering the contributions of interactions on the early AP-1 response, our analysis reveals AP-1 activation at 20 minutes to be influenced by c-Fos reaction mechanisms directly (α > 0.5). This finding suggests that interventions in specific c-Fos reaction mechanisms would affect significantly the early AP-1 response dominated by the ppc-Fos:ppc-Jun AP-1 dimer without requiring a simultaneous intervention in multiple reaction mechanisms. In contrast, the AP-1 activation at intermediate or late times is influenced by c-Jun based reaction mechanisms with less direct effects (α < 0.5). This result suggests that affecting the intermediate and late AP-1 response, at which times ppc-Jun:ppc-Jun is activated significantly, would require interventions in multiple simultaneous c-Jun reaction mechanisms.

#### Relating AP-1 activity dynamic balance to key network mechanisms

Key network nodes are often identified on the basis of the magnitude of changes in experimentally measured node levels. However, identifying key network nodes on this basis may neglect to consider the class of network nodes that are influential even at low amounts. For example, experiments measuring the level of c-Fos mRNA commonly interpret this to be an indicator of neuronal activity that is dependent on Fos-mediated mechanisms. However, our results question this common assumption, and suggest an alternative perspective on the basis of sensitivity analysis in context with network connectivity. In the following, we present our global sensitivity analysis results in the context of changing network node levels. We develop an annotated network diagram that integrates the visualization of network interactions with node levels along and sensitivity data as a Dynamic Network Sensitivity and Interaction Map (DyNSIM). Following our visual analysis approach employing DyNSIM, we find that c-Fos mRNA dynamics alone fails to reflect the shifting sensitive reaction mechanisms underlying AT1R induced AP-1 activation. However, the levels of active form of c-Fos protein (ppc-Fos) do correlate with the shift in sensitivities in the system. The contextual relationships between species levels and sensitivities as revealed in DyNSIMs are not obvious from an analysis of the network structure or species levels alone. These were unraveled using the decision tree methods, detailed below.

To place our global sensitivity results in the context of AT1RGRN predictions, we developed a series of AT1RGRN DyNSIMs after 20, 40, and 60 minutes of Ang II treatment, as shown in Figures 5E, F, and 5G. Through such DyNSIM representation of our model predictions we find that the several network nodes present at persistently high levels do not contribute to the model-predicted AP-1 sensitivity, as detailed below.

After 20 minutes of Ang II treatment, ppc-Fos:ppc-Jun is predicted to be the dominant AP-1 dimer and, consequently, AP-1 is most sensitive to Fos reaction mechanisms (Figures 5B and 5E). By this point in AT1RGRN simulation, active ERK has phosphorylated Elk-1 to induce its transcriptional activity, initiating the synthesis of new c-Fos protein. Newly synthesized c-Fos protein is rapidly converted to phosphorylated c-Fos by FRK, which has maximal activity at this time, and thus available to form ppc-Fos:ppc-Jun AP-1 dimers. At the same time, the level of c-Jun in different phosphorylation states is high, potentially suggesting a prominent role of Jun reaction mechanisms on AP-1 activation as well. However, model simulations predict this fully phosphorylated c-Jun to be present as ppc-Jun:ppATF-2 leading to a delayed increase in c-Jun synthesis rather than an immediate impact on AP-1 activation at this time.

The predicted AT1RGRN response at 40 minutes is characterized by an equal contribution of ppc-Fos:ppc-Jun and ppc-Jun:ppc-Jun in activated AP-1 (Figures 5C and 5F). At this time, the sensitivity of AP-1 has shifted away from Fos based reaction mechanisms to that of Jun based mechanisms related to c-Jun synthesis and JNK signaling. These network parameters arise as critical at this time frame because of their role in determining the activity of the positive feedback loop controlling the synthesis of c-Jun. At this time, the levels of c-Fos protein and gene expression remain high, but decreasing FRK activity limits the conversion of c-Fos to ppc-Fos, and thus attenuates the formation of ppc-Fos:ppc-Jun. Fully phosphorylated c-Jun has begun to accumulate at this time as a result of newly synthesized c-Jun protein being phosphorylated by sustained JNK activity.

After 60 minutes of Ang II treatment, ppc-Jun:ppc-Jun is predicted to be the dominant AP-1 dimer and the sensitivity of AP-1 has fully shifted to Jun mechanisms (Figures 5D and 5G). However, the levels of c-Fos protein and mRNA remain high, though an absence of FRK activity has ceased c-Fos phosphorylation and formation of ppc-Fos:ppc-Jun. An abundance of fully phosphorylated c-Jun allows the formation of dominant ppc-Jun:ppc-Jun AP-1 dimers.

These contextual relationships between species levels and sensitivities as revealed in the series of DyNSIMs provides insights into the predicted shift in sensitivity of AP-1 activity from Fos:Jun heterodimer based mechanisms to that of Jun homodimer based reactions over time. Additionally, the DyNSIM representation reveals a lack of strong instantaneous correlation between the level of network nodes that are routinely measured experimentally (such as c-Fos mRNA, c-Fos protein, c-Jun mRNA, and c-Jun protein) and their related reaction mechanisms. For instance, although c-Fos mRNA remains high throughout AT1RGRN simulation, the composition of AP-1 has shifted away from ppc-Fos:ppc-Jun to ppc-Jun:ppc-Jun after 40 minutes of network activation. The rate-limiting factor by this timeframe for additional c-Fos activation is not the c-Fos mRNA or protein, but the upstream kinase FRK. Our sensitivity analysis captures this dependence and hence yields low instantaneous correlation between computed sensitivities and observed mRNA and protein levels for c-Fos in this time frame. In contrast, our analysis reveals AP-1 DNA binding activity dynamics to be highly sensitive to the maximum concentration of JNK at the end of AT1RGRN simulation, despite the activity of JNK having returned to basal activity after reaching a peak activity at 30 minutes. This low instantaneous correlation between the AP-1 sensitivity and JNK levels is unexpected on the surface, as contrasted with the result for FRK and c-Fos dependent mechanisms at 20 and 40 min time point. However, we note that by 60 min time point, the AP-1 composition primarily shifted to that of c-Jun homodimer, and the level of c-Jun at this time is dependent on the peak activity of JNK and not instantaneous levels (due to multiple processes leading from JNK to c-Jun protein, yielding a higher order dynamic system). This contrasting temporal correlation between sensitivities and network node levels measured experimentally and AP-1 sensitivity further highlights the complexity of this gene regulatory network.

#### Relating Network Perturbations to Distinct Classes of AP-1 Activity Dynamics

We explored the effect of variations in the seven most significant network parameters, the dynamics of which are shown in Figure 5A, on AP-1 response profiles as categorized into three classes (ppc-Fos: ppc-Jun dominated, Dynamic balance, and ppc-Jun: ppc-Jun dominated). We followed a multivariate perturbation and decision tree analysis approach similar to that pursued by [37]. We (1) generated a large number of perturbed parameter sets (~1 million), (2) simulated the AP-1 DNA binding activity dynamics in each case, (3) classified the AP-1 response as belonging into one of the three classes, and (4) related the perturbations in network parameters to the classes of AP-1 response through construction of a decision tree. A detailed description of our multivariate approach is provided in the Methods section.

**Figure 6:**
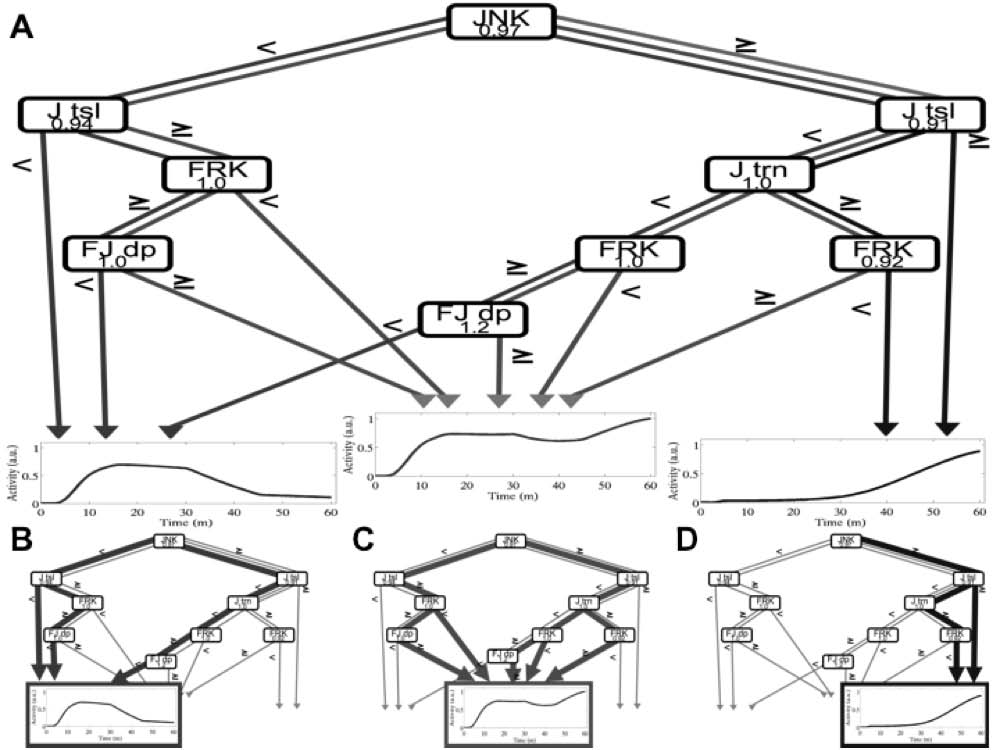
**Decision-tree analysis**. **(A)** Decision-tree relating perturbations in model parameters and their hierarchical dependencies to potential Total AP-1 activity responses. **(B-D)** Specific perturbations leading to **(B)** ppc-Fos:ppc-Jun alone **(C)** dynamic balance, and **(D)** ppc-Jun:ppc-Jun alone. Tree branches represent perturbed parameters, while their edges indicate the magnitude of perturbation. Terminal leaves represent the simulated Total AP-1 activity profile corresponding to ppc-Fos:ppc-Jun alone (left), ppc-Jun:ppc-Jun alone (right), and ppc:Fos:ppc-Jun + ppc-Jun:ppc-Jun (middle).

The decision tree presented in Figure 6 reveals the predicted dynamic balance of AP-1 activation to be robust to perturbations in individual network parameters. Realizing an AP-1 outcome deviating from the dynamic balance requires specific simultaneous perturbations in multiple parameters. For instance, significant increase in JNK activity in conjunction with an increase in the rate of c-Jun translation would lead to a ppc-Jun:ppc-Jun AP-1 response, even in the face of changes to other network parameters, while perturbations in either reaction alone is insufficient to cause a deviation from the dynamic balance response. Similarly, a simultaneous decrease in JNK activity and a decrease in c-Jun translation would lead to AP-1 DNA binding activity dynamics resembling ppc-Fos:ppc-Jun activity dynamics.

Network parameters related to the maximal activation of JNK, which was identified to be the most influential on AP-1 activity via global sensitivity analysis, is consistently the key indicator of AP-1 network outcome. Specifically, total AP-1 responses resembling the activation of ppc-Jun:ppc-Jun are only possible through an increase in JNK signaling, while total AP-1 responses resembling the activation of ppc-Fos:ppc-Jun are only possible when JNK signaling is decreased.

Figure 6 highlights the different paths representing particular parameter subdomains that lead to distinct classes of AP-1 dynamical response. For instance, higher levels of JNK and Jun translation lead to Jun homodimer dominated response, whereas lower levels of JNK coupled with lower Jun translation lead to a Fos-Jun heterodimer dominated dynamics. These paths are subtly altered by FRK levels in a predictable fashion with lower FRK corresponding to Jun homodimer dynamics and higher FRK level corresponding to that Fos-Jun heterodimer response. Interestingly, a majority of the paths lead to the dynamic balance indicating the robustness of this model-predicted AP-1 activity response.

## Conclusions

In this paper we develop novel computational approaches and a model to analyze and represent the TH gene regulatory network orchestrated by AT1R activation in the brainstem. By developing this model, the first to describe this network that plays a critical role in blood pressure regulation, we are able to use model simulation and analysis to provide quantitative insight into network dynamics inaccessible by experimental approaches. We reveal the dynamics of AP-1 activation to be comprised of individual AP-1 TF dimers operating at distinct times to modulate TH gene expression. We predict that TH gene expression is induced through a regulatory network employing parallel reaction mechanisms to activate a dynamic balance of AP-1 TF dimers. These results describe an integrative process by which the AT1R gene regulatory network activates AP-1 robustly in the presence of perturbations in network reaction mechanisms present in heterogeneous brain tissue. This hypothesis is supported by our application of a novel multivariate analysis approach that found AP-1 activation to be sensitive to a small number of reaction mechanisms. The activation of AP-1 as a dynamic balance of TF dimers remains the model outcome in a majority of perturbed parameters.

Our results show that c-Fos gene expression, which is widely used as an indicator of neuronal activity, needs to be interpreted with caution based on the time scales of interest, as it may not be a true indicator of c-Fos:c-Jun in the dimer composition of activated AP-1 over long time periods of neuronal stimulation. Within the context of the mechanisms considered in the model, the time profile of c-Fos mRNA levels do not correlate with the shift in the sensitivities, as the mRNA continues to be at higher levels even as the AP-1 composition and sensitivities shifted. However, our analysis also reveals that the active form of c-Fos protein directly correlates with the shift in the sensitivities in the system (Figure 5).

### Experimental Context: AT1R-activated Gene Regulatory Network

We here use computational modeling in order to understand integrative mechanisms suggested by our own experimental data and that appear complex and unexplained in the literature. Our transcript profiling time-series experiments indicated a role of AT1R and AP-1 in the in vivo NTS response to acute hypertension (Khan et al., 2008). As a means of exploring the dynamics of the AT1R-activated gene regulation, we developed a computational model describing the downstream activation of the AP-1 family of TFs and their regulation of TH gene expression. Our model takes its structure from the network topology suggested by molecular biology experiments conducted using cultured neurons [17,19,24,35]. Data from these previous studies raise questions showing apparently conflicting data as to the possible role of various kinases in the AT1R response. In addition, these studies cannot determine the role of interactions and dynamics in the individually measured variables in the response process.

### Dynamical Systems Modeling to Explore Interactions in AT1R Gene Regulatory Network

We here present a dynamic computational model of the AT1R gene regulatory network to achieve our specific goals of studying the interactions in the network. The model focuses on functional issues within the domain of AT1R responses in neurons. It describes how the kinases, transcription factors and genes involved in the response are integrated into a collective process. Our analysis of the model provides an understanding of the system requirements for robust function by uncovering the role of specific reactions operating in a physiologically plausible range of parameters.

Our specific network model uses reaction mechanisms and assumptions that have been made in similar models of gene regulation networks [29,32] and protein phosphorylation [30,31]. Based on model simulations, we developed hypotheses on a dynamic shift of AP-1 TF dimer composition, from a c-Fos and c-Jun heterodimer to that of a c-Jun homodimer, as underlying the total AP-1 response. This model prediction of dynamically switching AP-1 dimers captures the experimentally observed overall AP-1 activity, and suggests a resolution to a previously confounding interpretation of experiments using pathway inhibitors (Figure 4). This prediction may be validated experimentally using FRK signaling inhibitors or RNA interference to reduce the activity of FRK while measuring the effect on AT1R activation of AP-1. Furthermore, by predicting a network employing redundant mechanisms for AP-1 activation, our modeling results suggests the AT1R-modulated induction of TH gene may be robust to perturbations in network components. By employing a novel multivariate analysis approach, we support the hypothesis of robust AP-1 activation.

### Multivariate Analysis to Reveal Key Nodes and Mechanisms in the AT1R Gene Regulatory Network Response

A key advantage of representing biological networks with mathematical models lies in their amenability for identifying network properties using engineering analysis approaches. Sensitivity analysis, for instance, is routinely used to identify the key network parameters that shape model 'output' of interest [31,36,37]. In sensitivity analysis, the effect of variations in one network parameter on model output is evaluated in the absence of variations in all other network parameters. However, this approach neglects the effect of perturbations in multiple network parameters simultaneously, as may be found in heterogeneous tissue. In this work, we address the effect of variations in multiple reaction parameters simultaneously by using a multivariate analysis combining global sensitivity analysis with decision tree analysis.

Global sensitivity analysis reveals that most of the parameters do not significantly affect the AP-1 DNA binding activity dynamics indicating the robustness of our hypotheses on the dynamic balance of AP-1 dimer forms. Our analysis yielded a focused subset of seven key parameters (out of 77 total) with temporally varying sensitivities. We are able to interpret these parameter sensitivity profiles as differential dynamic contribution of Fos- and Jun-dependent mechanisms to AP-1 activity. We visualized these predictions in the context of network interactions and species levels using a novel approach (DyNSIMs in Figure 5). Based on these findings, we propose that the AT1R gene regulatory network ensures robust TH gene expression in the presence of network perturbations and cellular heterogeneity by utilizing a family of transcription factors whose individual components are operational at distinct times. This hypothesis is further supported by our analysis of the effect of variability in the network nodes on particular classes of AP-1 activation dynamics.

In order to approach this issue of robustness in the face of cellular heterogeneity, we analyzed the relationships between the seven key parameters using a multivariate perturbation and decision tree approach (Figure 6). This analysis indicated that while there may be a large combinatorial functional space feasible for neuronal states and parameters, the network behavior is constrained to a small set of AP-1 response profiles. Note that many of the paths through this space lead to a dynamic balance of AP-1 dimer forms, yielding a robust AP-1 response counteracting the biological variability. One consequence of this result is that the validation of dynamic AP-1 balance does not require a highly controlled cell line or similar model experimental systems, but would rather be applicable more generally and verifiable in the context of *in vivo *biological heterogeneity.

The absence of interactions among key parameters during early AP-1 activation suggests individual reaction mechanisms as potential control points for altering the immediate early phase of AP-1 activation dynamics. Our analysis predicts that influencing FRK activity to regulate early AP-1 activation would be effective in a way that is not critically dependent on the rest of the network parameters. Potential individual control points for longer-term AP-1 activation are less clear due to the significant presence of interactions among sensitive reaction mechanisms as identified by the global sensitivity analysis. Hence, manipulating the long-term AP-1 DNA binding activity dynamics would require simultaneous perturbations in multiple parameters, counteracting the robustness revealed in our multivariate perturbation approach.

### Future Extensions to a System-wide AT1R Gene Regulatory Network

The present gene regulatory network model lays the foundation for future modeling efforts to describe additional molecular processes involved in the NTS adaptation to the hypertensive state. Future work will focus on extending the description of the AT1R gene regulatory network to study the effects on system-wide transcription factors and genes towards a systems level understanding of this important response process. For example, extensions to the present model may include CREB and ATF family of TFs that were predicted as participating in the NTS response to AT1R activation and elevated blood pressure [20,21], to provide a more realistic description of the complex interplay between multiple TFs regulating their target gene expression. Another avenue for model expansion is 'upstream' to the present model in developing detailed quantitative description of AT1R mediated signaling pathways. Incorporating feedback of transcriptional and post-transcriptional regulation onto the signaling pathways and membrane electrical behavior will yield an integrated description that could be used to study long-term adaptive changes in the neurons. The multivariate analysis approach developed in this work may be applied broadly to other mathematical models of biological systems.

## Methods

### Simulating AT1RGRN

AT1RGRN describes the molecular events linking the binding of Ang II to AT1R in the brain to the induction of tyrosine hydroxylase gene expression through the activation of the AP-1 family of transcription factors. AT1RGRN is comprised of 29 ordinary differential equations with cytoplasmic and nuclear compartments. A reaction scheme of AT1RGRN, the mass balances of model species is given in Additional file 1, Table S1, and the model reaction rates are given in the Additional file 1. AT1RGRN was simulated in the MATLAB computing environment (The Mathworks, Natick, MA) using the *ode15s *numerical integration algorithm. Integration steps were taken at a fixed interval of every minute between 0 and 60 minutes after simulated Ang II treatment.

The presented model uses the experimentally measured dynamics of ERK, FRK, and JNK as model input, relating an experimental addition of 100 nM Ang II. Model inputs are linearly interpolated from experimental data measured between 0 and 60 minutes after Ang treatment [26,28]. The dynamics of AT1RGRN inputs are given in the Additional file 1, and the conversion between experimentally measured kinase dynamics and AT1RGRN model inputs is described in Normalization below.

### Normalization

In order to compare the dynamics of model species predicted by AT1RGRN to the dynamics measured experimentally, we normalized experimental data and model predictions to dimensionless units. Dynamic experimental data was made dimensionless with an initial value of zero and maximum value of one by (1) subtracting the experimental measurement at time zero from all data points, and (2) dividing the experimental measurements by the maximum value from Step (1). The time-series responses of model species were normalized in the same fashion to be dimensionless with an initial value of zero and maximum value of one. The nuclear concentrations of input kinases were calculated by scaling the normalized dynamics measured experimentally by scaling factors that corresponded to the nuclear concentration at maximal stimulation.

### Fitting Model Parameters

We began fitting the reaction parameters in AT1RGRN by using initial estimates of parameter values from similar models available in the literature.

• Initial estimates of parameters describing protein phosphorylation (reactions 1, 4, 8, 10, 13, 17, and 19) and dephosphorylation (reactions 2, 5, 9, 11,14, 18, 20, 23, 26, and 28) were obtained from [30].

• Initial estimates of kinetic parameters describing gene transcription (reactions 30, 37, 44, and 46), protein synthesis (reactions 34 and 41), protein degradation (reactions 3, 6, 7, 12, 15, 16, 22, and 25), and mRNA degradation (reactions 33, 40, and 48) were taken from [29].

• Initial estimates of parameters characterizing AP-1 dimerization (reactions 21, 24, and 27), protein:DNA binding (reactions 29, 36, 43, and 45), pre-mRNA splicing and processing (reactions 32, 39, and 47), and protein translocation (reactions 35 and 42) were found in [32].

• The basal rate of transcription of c-Fos (reaction 31) and c-Jun (reaction 38) were initially estimated as 1/1000 the maximum rate of transcription taken from [29].

• Cytoplasmic and nuclear diameters of brainstem neurons were estimated by confocal microscopy images published in Figure 3A of [40], which allowed computation of cellular and nuclear volumes by assuming spherical geometry and taking cytoplasmic volume as the difference between cellular and nuclear volumes. This gave nuclear and cytoplasmic volumes estimates of 14.1 × 10^-9 ^μL and 65.3 × 10^-9 ^μL, respectively.

• The initial concentrations of all model species, with the exception of unphosphorylated ATF-2 and Elk-1, were estimated as zero and excluded from parameter fitting. Unphosphorylated ATF-2 and Elk-1 were estimated to be present in the nucleus at concentrations of 56.312 nM.

• The maximum concentration of ERK, FRK, and JNK were estimated as 100 nM, and excluded from model fitting.

• Compartmental volumes, maximum concentrations of AT1R-activated kinases, initial concentrations of ATF-2 and Elk-1, and the initial concentration of unoccupied gene promoters, taken as two unoccupied binding sites per nuclear volume, were not included in parameter fitting and were fixed to their initial estimates.

It should be noted that a model with such a large number of parameters and complex feedback and cross-talk mechanisms could potentially yield a number of dynamic profiles of AP-1 activity. Hence, we follow a step-wise approach to constrain the model behavior to that of experimental observations from brainstem neurons, as detailed below.

Generally, model parameters were fit by minimizing the sum-squared error between the model predicted responses of neurons stimulated by 100 nM Ang II and the experimentally measured responses. Model predictions and experimental data were made comparable through normalization procedures, as described below. The model-predicted nuclear concentration of pElk-1 was compared to the DNA-binding activity published in [33]. Similarly, the model-predicted cytoplasmic concentration of c-Fos mRNA was compared to the c-Fos mRNA data published in [34]. The sum of nuclear concentration of ppc-Fos:ppc-Jun and ppc-Jun:ppc-Jun as predicted by AT1RGRN was compared to the AP-1 DNA-binding activity published in [35]. Finally, the cytoplasmic concentration of TH mRNA was compared to the experimentally measured TH gene expression published in [28].

Our approach to fit model parameters began by generating a large dataset of perturbed model parameters. Each of the 6 × 10^6 ^rows in this dataset contained a set of all of the 72 model parameters that were randomly perturbed from their initial estimates. Model simulations were then performed for each set of perturbed model parameters to predict the dynamics of pElk-1, c-Fos mRNA, AP-1, and TH mRNA. This allowed the calculation of sum squared error between these model predicted dynamics and the experimentally measured dynamics for each set of perturbed parameters. We then identified candidate parameter sets by selecting those that produced model output that matched the experimental data at various levels of the AT1R activated gene regulatory network. Specifically, we selected model parameters from 6 × 10^6 ^potential parameter sets by first limiting to those that fit the dynamics of pELK-1, then further reduce the size of our perturbed parameter set by imposing the constraint of matching c-Fos mRNA dynamics. Next, we imposed additional constraints fitting the experimentally observed AP-1 DNA binding activity dynamics, and then concluding with the constraint that model predictions match the TH mRNA dynamics. The result of our model fitting procedure is shown in Additional file 1, Figure S1.

### Implementing Global Sensitivity Analysis

The global sensitivity analysis used in this work essentially followed the procedure described in [39]. Briefly, the total-effects sensitivity, first-order sensitivity, and second-order sensitivity of total AP-1 were calculated for all 77 parameters in AT1RGRN. AT1RGRN parameters include 72 reaction rate parameters, the non-zero initial conditions of ATF-2 and Elk-1, and the maximum concentrations of input kinases FRK, ERK, and JNK.

The foundation of this approach is to estimate the variance of AT1RGRN predicted AP-1 output, then to decompose this variance into the contributions given by each model parameter acting individually, in addition to contributions made through interactions between parameters operating together to affect AP-1. Taking this approach, we decompose the variance of AP-1 as

(1) $$V\left(\text{AP}-\text{1}\right)=\sum_{i}V_{i}+\sum_{i}\sum_{j>i}V_{ij}+\ldots +V_{12\ldots k}$$

where *i *∈ 1 ... *k*, the index corresponds to the set of perturbed parameters. In our analysis, we investigated the sensitivity of model output to 77 model parameters. From this, we estimate the total effect of a given parameter (S__T__) and its direct effect in the absence of interactions with other parameters, which is its first-order sensitivity (S__1__)

(2) $$S_{T}^{i}=\frac{V_{i}+\sum_{j>i}V_{ij}+\ldots +V_{12\ldots k}}{V\left(\text{AP}-\text{1}\right)}$$

(3) $$S_{1}^{i}=\frac{V_{i}}{V\left(\text{AP}-\text{1}\right)}$$

These sensitivity indices, $S_{T}^{i}$ and $S_{1}^{i}$, refer to the effect of a given model parameter *i *∈ 1 ... *k*, on the output of interest. However, for clarity, we omit the superscript in the text when we refer to these indices in the context of the corresponding model parameter. We followed a numerical Monte Carlo simulation approach detailed in [39] to estimate the sensitivities given above. We considered parametric perturbations within a two-fold range, such that $\frac{p_{b}}{2}\leq p\leq 2*p_{b}$, where *p *and *p*_*b *_are the set of perturbed and base parameters, respectively. These sensitivity coefficients were calculated for each of the 60 minutes of simulated AP-1 activation. Parameter values were perturbed within 2-fold of their nominal values by applying the 'MatousekAffineOwen' algorithm to scramble a set of 77-dimensional pseudo-random numbers generated from the Sobol sequence in the [41]. A sample size of 1 × 10^5 ^simulations was used to estimate the variance of total AP-1 used in global sensitivity analysis calculations. Sensitivity coefficients calculated when the variance of AP-1 was less then 10% the maximum variance of AP-1 were excluded from further analysis to avoid numerical oddities, as shown in the Additional file 1, Figure S2.

By following the described global sensitivity analysis approach, we are able to estimate the effect of interactions among AT1RGRN reaction mechanisms on the predicted AP-1 response. We quantify the presence of interactions on *i*^th ^reaction mechanism's influence by taking the following ratio

(4) $$\alpha^{i}=\frac{S_{1}^{i}}{S_{T}^{i}}$$

where 0 ≤ α^*i *^≤ 1. As before, for clarity, we drop the supserscript *i *in subsequent text as we discuss the computed ratios in the context of the corresponding parameters. A value of α near one indicates that the influence of that particular reaction mechanism on AP-1 is not significantly conditional upon other parameter values. Conversely, α value approaching zero indicates that the particular reaction mechanism exerts its influence exclusively through interactions with other AT1RGRN parameters.

### Decision Tree Analysis Methodology

Our decision tree methodology began by generating 1 × 10^6 ^sets of perturbed parameters in the seven most influential model parameters as identified by the global sensitivity analysis. Parameter values were perturbed within 2-fold of their nominal values by applying the 'MatousekAffineOwen' algorithm to scramble a set of 7-dimensional pseudo-random numbers generated from the Sobol sequence in the MATLAB computational environment [41]. Total AP-1 activity dynamics for each perturbed parameter set were then calculated by simulating AT1RGRN, and the AP-1 response was classified using the template matching procedure of Pavlidis et al [42]. Total AP-1 activity dynamics predicted by each set of perturbed parameters were compared to the following three templates: the nominal dynamics of ppc-Fos:ppc-Jun, the nominal dynamics of ppc-Jun:ppc-Jun, and the nominal dynamics of total AP-1. A Pearson's product moment correlation coefficient greater than 0.95 was used to classify the perturbed AP-1 response as resembling the nominal ppc-Jun:ppc-Jun response, the nominal ppc-Jun:ppc-Jun response, or the nominal dynamics of total AP-1. Parameter sets that failed to meet this criterion were excluded from further classification. Relation of AP-1 outcome to perturbed parameter values was performed using the 'rpart' library in the R statistical computing environment [43]. In similar fashion to the decision tree analysis employed by [37], we imposed a constraint for decision tree construction that a node can only be split again if it contained at least 15% of the smallest AP-1 response category.

## Authors' contributions

GMM implemented the network model in MATLAB, performed the simulations and analysis, and drafted the manuscript. BAO and JSS assisted in the model development, in interpretation of the results and in preparing the manuscript. RV supervised the study, formulated the mathematical model, designed the combination of sensitivity and decision-tree analysis methods, assisted in interpretation and presentation of the results, and drafted the manuscript. All authors read and approved the final manuscript.

## Supplementary Material

Additional file 1 **Supplementary figures and tables**. The file contains the following figures and tables: Figure S1 - Dynamics of Input Kinases and Comparison of AT1RGRN Model Prediction with Experimental Data. Figure S2 - Variance of AT1RGRN-predicted AP-1. Figure S3 - Total-effects and First-Order Sensitivities of AT1RGRN-predicted AP-1. Table S1 - Model Reactions. Table S2 - Mass Balances. Table S3 - AT1RGRN Parameters. Table S4 - Parameter Index Corresponding to Figure S4. Click here for file
