# Supplementary material for: Robust dynamic balance of AP-1 transcription factors in a neuronal gene regulatory network
Source: BMC Syst Biol. 2010 Dec 17;4:171. doi: 10.1186/1752-0509-4-171 (PMC3019179; doi:10.1186/1752-0509-4-171)
Supplement: Additional file 1 — Supplementary figures and tables. The file contains the following figures and tables: Figure S1 - Dynamics of Input Kinases and Comparison of AT1RGRN Model Prediction with Experimental Data. Figure S2 - Variance of AT1RGRN-predicted AP-1. Figure S3 - Total-effects and First-Order Sensitivities of AT1RGRN-predicted AP-1. Table S1 - Model Reactions. Table S2 - Mass Balances. Table S3 - AT1RGRN Parameters. Table S4 - Parameter Index Corresponding to Figure S4. [file 1752-0509-4-171-S1.DOC]

**Supplementary Information**

**Robust dynamic balance of AP-1 transcription factors in an angiotensin II activated neuronal gene regulatory network**

Gregory M. Miller^1, 2^, Babatunde A. Ogunnaike^2^, James S. Schwaber^1^,

Rajanikanth Vadigepalli^1*^

^1^Daniel Baugh Institute for Functional Genomics and Computational Biology,

Department of Pathology, Anatomy and Cell Biology, Thomas Jefferson University

Philadelphia, PA, USA 19107

^2^Department of Chemical Engineering, University of Delaware

Newark, DE, USA 19716

*Corresponding Author: [raj@mail.dbi.tju.edu](mailto:raj@mail.dbi.tju.edu)

**Contents**

A. Supplementary Figures

S1. Dynamics of Input Kinases and Comparison of AT1RGRN Model Prediction with Experimental Data

S2. Variance of AT1RGRN-predicted AP-1

S3. **Total-effects and First-Order Sensitivities** of AT1RGRN-predicted AP-1

B. Supplementary Tables

S1. Model Reactions

S2. Mass Balances

S3. AT1RGRN Parameters

S4. Parameter Index Corresponding to Figure S4

**A. Supplementary Figures**


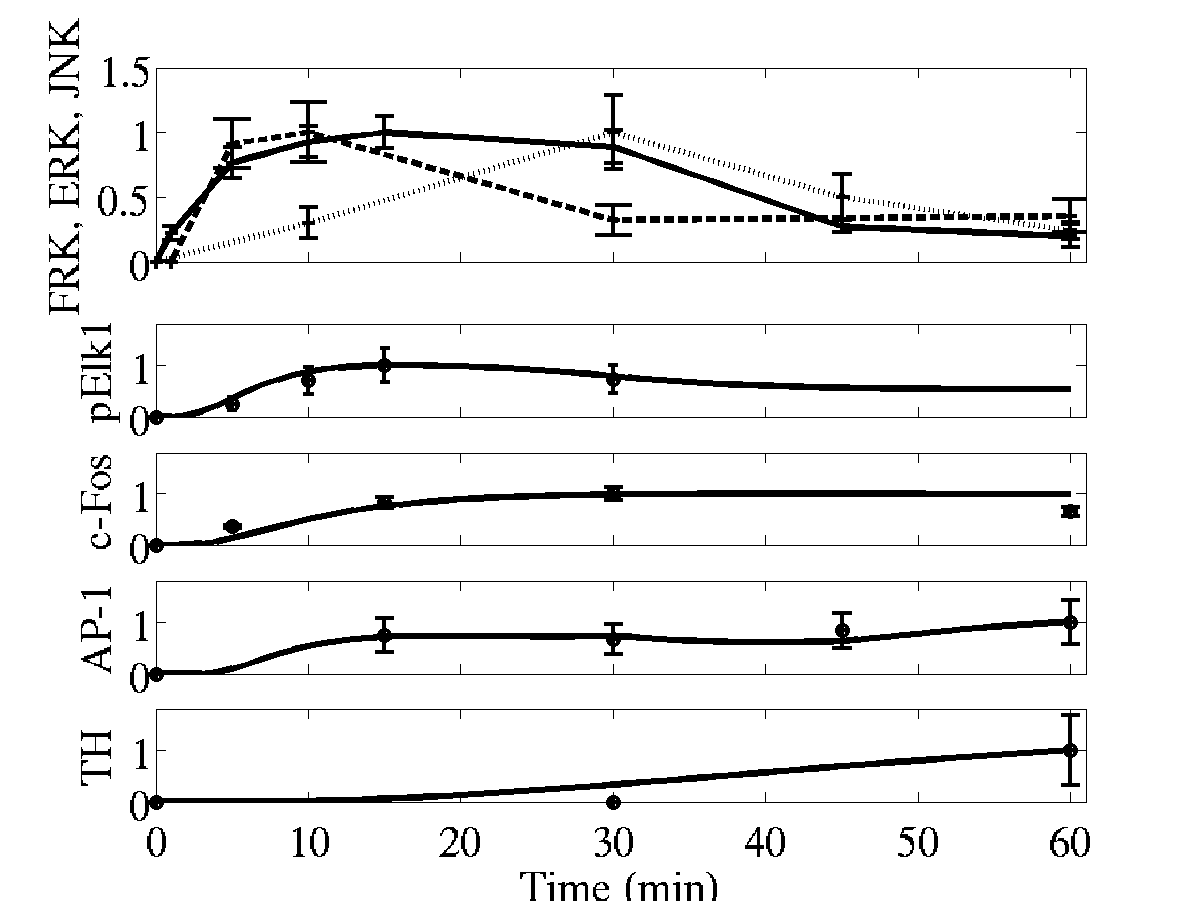


Figure S1: Dynamics of Input Kinases and Comparison of AT1RGRN Model Prediction with Experimental Data. First panel: The dynamics of AT1R-induced input kinases FRK (Solid), ERK (Heavy-dashed), JNK (Light-dashed). Parameter values were fit to match the model predictions (lines) with experimental profiles (points) of pELk-1 (2nd Panel), c-Fos mRNA (3rd Panel), total AP-1 (4th Panel), and TH mRNA (5th Panel) as reported in the literature. All species levels are presented here in normalized dimensionless units resulting from normalizing data to have a zero initial value and maximum value of one. A description of the experimental data presented here is given in the Materials and Methods of the main text.


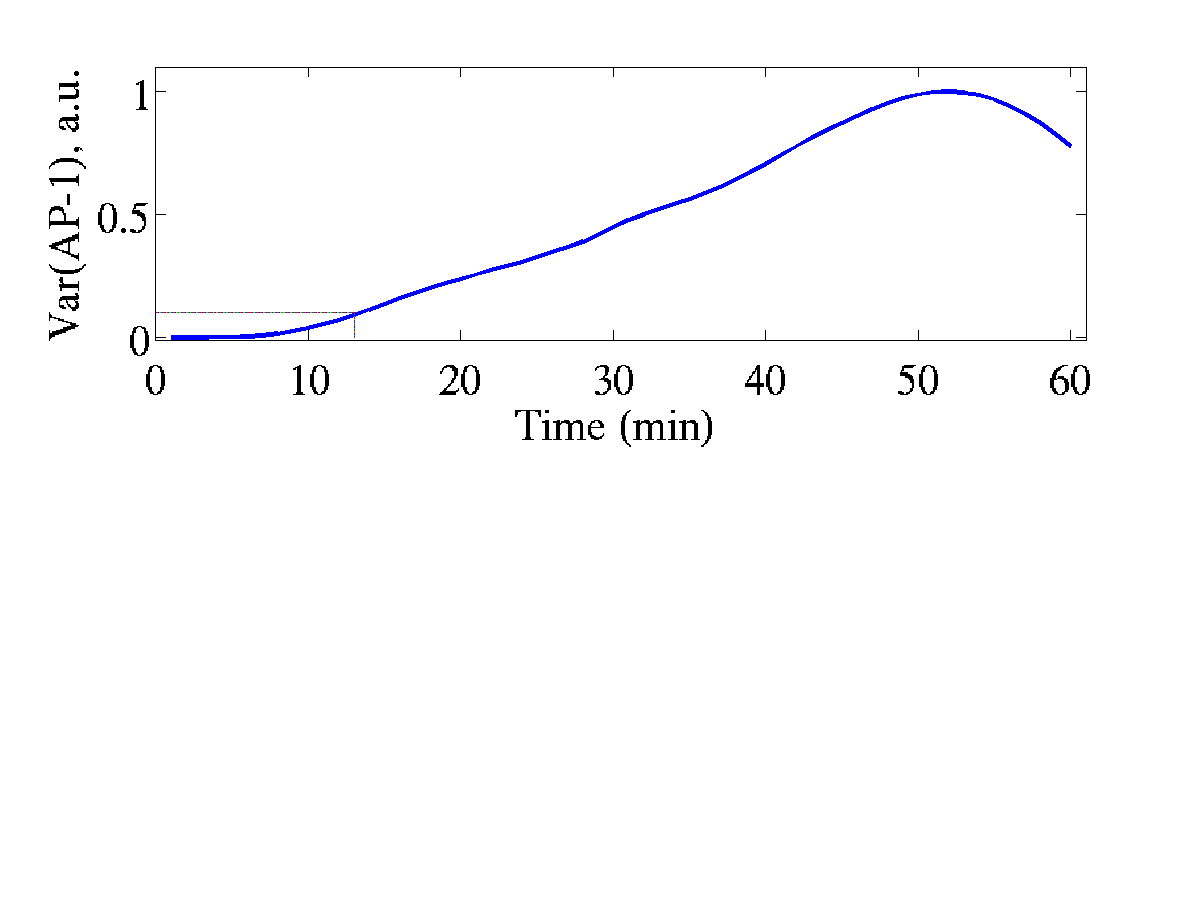


Figure S2: Variance of AP-1 (taken as the sum of ppc-Fos:ppc-Jun and ppc-Jun:ppc-Jun) as estimated by the Monte-Carlo simulation of AT1RGRN using 1 x 10^5^ sets of perturbed model parameters. Variance of AP-1 is presented here in normalized dimensionless units by dividing the variance at all time points by the maximum estimated variance of AP-1. The horizontal and vertical lines have been drawn to illustrate the threshold by which the variance of AP-1 exceeded 10% of the maximum estimated variance of AP-1 at any time. Global sensitivity analysis was performed during 14-60 minutes of Ang II treatment when the variance of AP-1 exceeded this threshold to avoid numerical oddities resulting from estimating sensitivities at times of low AP-1 variance. A description of the Monte-Carlo simulation procedure, and the global sensitivity analysis employed is given in the Materials and Methods of the main text.


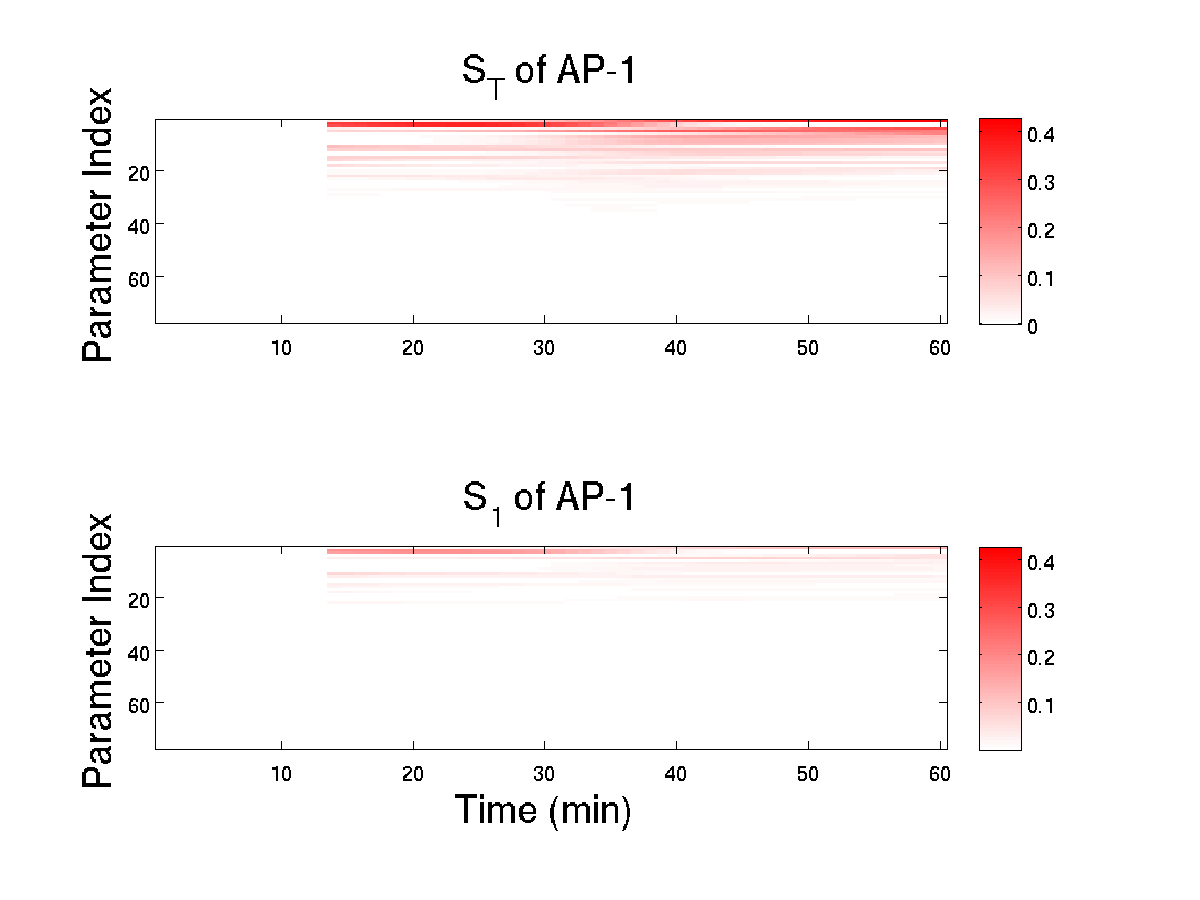


Figure S3: Heatmaps of total-effects (S_T_) and first-order (S_1_) sensitivities of AT1RGRN reaction parameters and initial conditions on Total AP-1, the sum of ppc-Fos:ppc-Jun and ppc-Jun:ppc-Jun. Global sensitivity analysis was performed during 14-60 minutes of Ang II treatment when the variance of AP-1 exceeded a threshold to avoid numerical oddities resulting from estimating sensitivities at times of low AP-1 variance, as described in Figure S1. A description of the AT1RGRN Reaction parameters and initial conditions corresponding to the Parameter Index is given in Table S4.

**B. Supplementary Tables**

**Table S1: Model Reactions**

| **Reaction Number** | **Reaction Rate** |
| --- | --- |
| **1** | $v_{1}=\frac{k_{1}[FRK][c-Fos]}{K_{1}+[c-Fos]}$ |
| **2** | $v_{2}=\frac{V_{2}[pc-Fos]}{K_{2}+[pc-Fos]}$ |
| **3** | $v_{3}=k_{3}[c-Fos]$ |
| **4** | $v_{4}=\frac{k_{4}[FRK][pc-Fos]}{K_{4}+[pc-Fos]}$ |
| **5** | $v_{5}=\frac{V_{5}[ppc-Fos]}{K_{5}+[ppc-Fos]}$ |
| **6** | $v_{6}=k_{6}[pc-Fos]$ |
| **7** | $v_{7}=k_{7}[ppc-Fos]$ |
| **8** | $v_{8}=\frac{k_{8}[ERK][Elk-1]}{K_{8}+[Elk-1]}$ |
| **9** | $v_{9}=\frac{V_{9}[pElk-1]}{K_{9}+[pElk-1]}$ |
| **10** | $v_{10}=\frac{k_{10}[JNK][c-Jun]}{K_{10}+[c-Jun]}$ |
| **11** | $v_{11}=\frac{V_{11}[pc-Jun]}{K_{11}+[pc-Jun]}$ |
| **12** | $v_{12}=k_{12}[c-Jun]$ |
| **13** | $v_{13}=\frac{k_{13}[JNK][pc-Jun]}{K_{13}+[pc-Jun]}$ |
| **14** | $v_{14}=\frac{V_{14}[ppc-Jun]}{K_{14}+[ppc-Jun]}$ |
| **15** | $v_{15}=k_{15}[pc-Jun]$ |
| **16** | $v_{16}=k_{16}[ppc-Jun]$ |
| **17** | $v_{17}=\frac{k_{17}[JNK][ATF-2]}{K_{17}+[ATF-2]}$ |
| **18** | $v_{18}=\frac{V_{18}[pATF-2]}{K_{18}+[pATF-2]}$ |
| **19** | $v_{19}=\frac{k_{19}[JNK][pATF-2]}{K_{19}+[pATF-2]}$ |
| **20** | $v_{20}=\frac{V_{20}[ppATF-2]}{K_{20}+[ppATF-2]}$ |
| **21** | $v_{21}=k_{21}[ppc-Fos][ppc-Jun]-k_{-21}[c-Fos:c-Jun]$ |
| **22** | $v_{22}=k_{22}[c-Fos:c-Jun]$ |
| **23** | $v_{23}=\frac{V_{23}[c-Fos:c-Jun]}{K_{23}+[c-Fos:c-Jun]}$ |
| **24** | $v_{24}=k_{24}[ppc-Jun]^{2}-k_{-24}[c-Jun:c-Jun]$ |
| **25** | $v_{25}=k_{25}[c-Jun:c-Jun]$ |
| **26** | $v_{26}=\frac{V_{26}[c-Jun:c-Jun]}{K_{26}+[c-Jun:c-Jun]}$ |
| **27** | $v_{27}=k_{27}[ppATF-2][ppc-Jun]-k_{-27}[c-Jun:ATF-2]$ |
| **28** | $v_{28}=\frac{V_{28}[c-Jun:ATF-2]}{K_{28}+[c-Jun:ATF-2]}$ |
| **29** | $v_{29}=k_{29}[c-Fos\_pr][pElk-1]-k_{-29}[c-Fos\_pr:pElk-1]$ |
| **30** | $v_{30}=k_{30}[c-Fos\_pr:pElk-1]$ |
| **31** | $v_{31}=R_{31}$ |
| **32** | $v_{32}=k_{32}\frac{V_{N}}{V_{C}}[c-Fos\_pre\_mRNA]$ |
| **33** | $v_{33}=k_{33}[c-Fos\_mRNA]$ |
| **34** | $v_{34}=k_{34}[c-Fos\_mRNA]$ |
| **35** | $v_{35}=k_{35}[c-Fos\_cyt]$ |
| **36** | $v_{36}=k_{36}[c-Jun\_pr][c-Jun:ATF-2]-k_{-36}[c-Jun\_pr:c-Jun:ATF-2]$ |
| **37** | $v_{37}=k_{37}[c-Jun\_pr:c-Jun:ATF-2]$ |
| **38** | $v_{38}=R_{38}$ |
| **39** | $v_{39}=k_{39}\frac{V_{N}}{V_{C}}[c-Jun\_pre\_mRNA]$ |
| **40** | $v_{40}=k_{40}[c-Jun\_mRNA]$ |
| **41** | $v_{41}=k_{41}[c-Jun\_mRNA]$ |
| **42** | $v_{42}=k_{42}[c-Jun\_cyt]$ |
| **43** | $v_{43}=k_{43}[TH\_pr][c-Jun:c-Jun]-k_{-43}[TH\_pr:c-Jun:c-Jun]$ |
| **44** | $v_{44}=k_{44}[TH\_pr:c-Jun:c-Jun]$ |
| **45** | $v_{45}=k_{45}[TH\_pr][c-Fos:c-Jun]-k_{-45}[TH\_pr:c-Fos:c-Jun]$ |
| **46** | $v_{46}=k_{46}[TH\_pr:c-Fos:c-Jun]$ |
| **47** | $v_{47}=k_{47}\frac{V_{N}}{V_{C}}[TH\_pre\_mRNA]$ |
| **48** | $v_{48}=k_{48}[TH\_mRNA]$ |

**Table S2: Mass Balances**

| **Species** | **Compartment** | **Mass Balance** |
| --- | --- | --- |
| c-Fos protein | Nucleus | $\frac{d}{dt}[c-Fos]=v_{2}+v_{23}+\frac{V_{C}}{V_{N}}v_{35}-v_{1}-v_{3}$ |
| pc-Fos protein | Nucleus | $\frac{d}{dt}[pc-Fos]=v_{1}+v_{5}-v_{2}-v_{4}-v_{6}$ |
| ppc-Fos protein | Nucleus | $\frac{d}{dt}[ppc-Fos]=v_{4}-v_{5}-v_{7}-v_{21}$ |
| Elk-1 protein | Nucleus | $\frac{d}{dt}[Elk-1]=v_{9}-v_{8}$ |
| pElk-1 protein | Nucleus | $\frac{d}{dt}[pElk-1]=v_{8}-v_{9}-v_{29}$ |
| c-Jun protein | Nucleus | $\frac{d}{dt}[c-Jun]=v_{11}+v_{23}+2v_{26}+v_{28}+\frac{V_{C}}{V_{N}}v_{42}-v_{10}-v_{12}$ |
| pc-Jun protein | Nucleus | $\frac{d}{dt}[pc-Jun]=v_{10}+v_{14}-v_{11}-v_{13}-v_{15}$ |
| ppc-Jun protein | Nucleus | $\frac{d}{dt}[ppc-Jun]=v_{13}-v_{14}-v_{16}-v_{21}-2v_{24}-v_{27}$ |
| ATF-2 protein | Nucleus | $\frac{d}{dt}[ATF-2]=v_{18}+v_{28}-v_{17}$ |
| pATF-2 protein | Nucleus | $\frac{d}{dt}[pATF-2]=v_{17}+v_{20}-v_{18}-v_{19}$ |
| ppATF-2 protein | Nucleus | $\frac{d}{dt}[ppATF-2]=v_{19}-v_{20}-v_{27}$ |
| ppc-Fos:ppc-Jun protein dimer | Nucleus | $\frac{d}{dt}[c-Fos:c-Jun]=v_{21}-v_{22}-v_{23}-v_{45}$ |
| ppc-Jun:ppc-Jun protein dimer | Nucleus | $\frac{d}{dt}[c-Jun:c-Jun]=v_{24}-v_{25}-v_{26}-v_{43}$ |
| ppc-Jun:ppc-ATF-2 protein dimer | Nucleus | $\frac{d}{dt}[c-Jun:ATF-2]=v_{27}-v_{28}-v_{36}$ |
| c-Fos promoter | Nucleus | $\frac{d}{dt}[c-Fos\_pr]=-v_{29}$ |
| c-Fos promoter:pElk-1 complex | Nucleus | $\frac{d}{dt}[c-Fos\_pr:pELK-1]=v_{29}$ |
| c-Fos pre-mRNA | Nucleus | $\frac{d}{dt}[c-Fos\_pre\_mRNA]=v_{31}+v_{30}-\frac{V_{C}}{V_{N}}v_{32}$ |
| c-Fos mRNA | Cytosol | $\frac{d}{dt}[c-Fos\_mRNA]=v_{32}-v_{33}$ |
| c-Fos protein | Cytosol | $\frac{d}{dt}[c-Fos\_cyt]=v_{34}-v_{35}$ |
| c-Jun promoter | Nucleus | $\frac{d}{dt}[c-Jun\_pr]=-v_{36}$ |
| c-Jun promoter:ppc-Jun:ppATF-2 complex | Nucleus | $\frac{d}{dt}[c-Jun\_pr:c-Jun:ATF-2]=v_{36}$ |
| c-Jun pre-mRNA | Nucleus | $\frac{d}{dt}[c-Jun\_pre\_mRNA]=v_{37}+v_{38}-\frac{V_{C}}{V_{N}}v_{39}$ |
| c-Jun mRNA | Cytosol | $\frac{d}{dt}[c-Jun\_mRNA]=v_{39}-v_{40}$ |
| c-Jun protein | Cytosol | $\frac{d}{dt}[c-Jun\_c]=v_{41}-v_{42}$ |
| TH promoter | Nucleus | $\frac{d}{dt}[TH\_pr]=-v_{43}-v_{45}$ |
| TH promoter:ppc-Jun:ppc-Jun complex | Nucleus | $\frac{d}{dt}[TH\_pr:c-Jun:c-Jun]=v_{43}$ |
| TH promoter:ppc-Fos:ppc-Jun complex | Nucleus | $\frac{d}{dt}[TH\_pr:c-Fos:c-Jun]=v_{45}$ |
| TH pre-mRNA | Nucleus | $\frac{d}{dt}[TH\_pre\_mRNA]=v_{44}+v_{46}-\frac{V_{C}}{V_{N}}v_{47}$ |
| TH mRNA | Cytosol | $\frac{d}{dt}[TH\_mRNA]=v_{47}-v_{48}$ |

**Table S3: AT1GRN Parameters**

| **Reaction Number** | **Parameters** |
| --- | --- |
| **1** | $k_{1}=0.154 s^{-1}$ $K_{1}=1.6189 nM$ |
| **2** | $V_{2}=8.4053 nM s^{-1}$ $K_{2}=6.8804 nM$ |
| **3** | $k_{3}=0.2741 s^{-1}$ |
| **4** | $k_{4}=5.1573 s^{-1}$ $K_{4}=3.0176 nM$ |
| **5** | $V_{5}=3.8786 nM s^{-1}$ $K_{5}=15.0773 nM$ |
| **6** | $k_{6}=1.1317 s^{-1}$ |
| **7** | $k_{7}=0.0730 s^{-1}$ |
| **8** | $k_{8}=0.2705 s^{-1}$ $K_{8}=65.2625 nM$ |
| **9** | $V_{9}=4.5337 nM s^{-1}$ $K_{9}=9.5340 nM$ |
| **10** | $k_{10}=6.5615 s^{-1}$ $K_{10}=27.1651 nM$ |
| **11** | $V_{11}=69.5154 nM s^{-1}$ $K_{11}=112.334 nM$ |
| **12** | $k_{12}=0.0214 s^{-1}$ |
| **13** | $k_{13}=1.3943 s^{-1}$ $K_{13}=2.069 nM$ |
| **14** | $V_{14}=11.9303 nM s^{-1}$ $K_{14}=110.6230 nM$ |
| **15** | $k_{15}=0.0053 s^{-1}$ |
| **16** | $k_{16}=0.00090396 s^{-1}$ |
| **17** | $k_{17}=0.4058 s^{-1}$ $K_{17}=18.5934 nM$ |
| **18** | $V_{18}=17.7672 nM s^{-1}$ $K_{18}=1.7933 nM$ |
| **19** | $k_{19}=1.3369 s^{-1}$ $K_{19}=21.5017 nM$ |
| **20** | $V_{20}=8.485 nM s^{-1}$ $K_{20}=3.3207 nM$ |
| **21** | $k_{21}=0.2867 nM^{-1} s^{-1}$ $k_{-21}=1.2753 s^{-1}$ |
| **22** | $k_{22}=0.0774 s^{-1}$ |
| **23** | $V_{23}=55.9578 nM s^{-1}$ $K_{23}=8.947 nM$ |
| **24** | $k_{-24}=428.219 s^{-1}$ $k_{24}=0.0546 nM^{-1} s^{-1}$ |
| **25** | $k_{25}=0.0498 s^{-1}$ |
| **26** | $V_{26}=105.164 nM s^{-1}$ $K_{26}=60.7342 nM$ |
| **27** | $k_{27}=0.9128 nM^{-1} s^{-1}$ $k_{-27}=1.7441 s^{-1}$ |
| **28** | $V_{28}=125.3660 nM s^{-1}$ $K_{28}=56.3667 nM$ |
| **29** | $k_{29}=3.4048 nM^{-1} s^{-1}$ $k_{-29}=3.1303 s^{-1}$ |
| **30** | $k_{30}=0.4274 s^{-1}$ |
| **31** | $R_{31}=\text{8.2285e-05} nM s^{-1}$ |
| **32** | $k_{32}=0.3316 s^{-1}$ |
| **33** | $k_{33}=0.1557 s^{-1}$ |
| **34** | $k_{34}=16.6871 s^{-1}$ |
| **35** | $k_{35}=\text{ 2.6297} s^{-1}$ |
| **36** | $k_{36}=0.2352 nM^{-1} s^{-1}$ $k_{-36}=14.6428 s^{-1}$ |
| **37** | $k_{37}=0.4267 s^{-1}$ |
| **38** | $R_{38}=\text{8.1524e-04} nM s^{-1}$ |
| **39** | $k_{39}=0.6621 s^{-1}$ |
| **40** | $k_{40}=0.068 s^{-1}$ |
| **41** | $k_{41}=59.9722 s^{-1}$ |
| **42** | $k_{42}=2.5531 s^{-1}$ |
| **43** | $k_{43}=0.0224 nM^{-1} s^{-1}$ $k_{-43}=4.3273 s^{-1}$ |
| **44** | $k_{44}=0.0042 s^{-1}$ |
| **45** | $k_{45}=1.1333 nM^{-1} s^{-1}$ $k_{-45}=0.6137 s^{-1}$ |
| **46** | $k_{46}=0.0042 s^{-1}$ |
| **47** | $k_{47}=0.0600 s^{-1}$ |
| **48** | $k_{48}=0.0031 s^{-1}$ |
| **Compartmental Volumes** | $V_{N}=14.1e-09 \mu L$ $V_{C}=65.3e-09 \mu L$ |
| **Non-zero Initial Conditions** | $[ATF-2]_{0}=28.156 nM$ $[Elk-1]_{0}=56.312 nM$ $[c-Jun\_pr]_{0}=[c-Fos\_pr]_{0}=[TH\_pr]_{0}=0.235 nM$ |
| **Maximum Nuclear Concentration of Input Kinases** | $[FRK]_{Max}=[ERK]_{Max}=[JNK]_{Max}=100.0 nM$ |

**Table S4: Parameter Index Corresponding to Figure S3**

| **Parameter Index** | **AT1RGRN Reaction Number** | **Parameter** |
| --- | --- | --- |
| **1** | Maximum Nuclear Concentration of Input Kinase | $[JNK]_{Max}$ |
| **2** | 23 | $V_{23}$ |
| **3** | Maximum Nuclear Concentration of Input Kinase | $[FRK]_{Max}$ |
| **4** | 13 | $k_{13}$ |
| **5** | 41 | $k_{41}$ |
| **6** | 26 | $V_{26}$ |
| **7** | 37 | $k_{37}$ |
| **8** | 36 | $k_{36}$ |
| **9** | 36 | $k_{-36}$ |
| **10** | 17 | $k_{17}$ |
| **11** | 23 | $K_{23}$ |
| **12** | 40 | $k_{40}$ |
| **13** | Non-zero Initial Condition | $[ATF-2]_{0}$ |
| **14** | 28 | $V_{28}$ |
| **15** | 38 | $R_{38}$ |
| **16** | 34 | $k_{34}$ |
| **17** | 28 | $K_{28}$ |
| **18** | 30 | $k_{30}$ |
| **19** | 26 | $K_{26}$ |
| **20** | 24 | $k_{24}$ |
| **21** | 24 | $k_{-24}$ |
| **22** | 12 | $k_{12}$ |
| **23** | 22 | $k_{22}$ |
| **24** | 18 | $V_{18}$ |
| **25** | 17 | $K_{17}$ |
| **26** | 19 | $k_{19}$ |
| **27** | 33 | $k_{33}$ |
| **28** | 19 | $K_{19}$ |
| **29** | 32 | $k_{32}$ |
| **30** | 25 | $k_{25}$ |
| **31** | 18 | $K_{18}$ |
| **32** | 39 | $k_{39}$ |
| **33** | 3 | $k_{3}$ |
| **34** | 7 | $k_{7}$ |
| **35** | 1 | $K_{1}$ |
| **36** | 10 | $k_{10}$ |
| **37** | 21 | $k_{21}$ |
| **38** | 20 | $V_{20}$ |
| **39** | 1 | $k_{1}$ |
| **40** | 8 | $k_{8}$ |
| **41** | 6 | $k_{6}$ |
| **42** | 42 | $k_{42}$ |
| **43** | 20 | $K_{20}$ |
| **44** | 21 | $k_{-21}$ |
| **45** | 4 | $K_{4}$ |
| **46** | 14 | $V_{14}$ |
| **47** | 16 | $k_{16}$ |
| **48** | 2 | $K_{2}$ |
| **49** | 29 | $k_{29}$ |
| **50** | 15 | $k_{15}$ |
| **51** | 9 | $K_{9}$ |
| **52** | Maximum Nuclear Concentration of Input Kinase | $[ERK]_{Max}$ |
| **53** | 8 | $K_{8}$ |
| **54** | 13 | $K_{13}$ |
| **55** | 11 | $V_{11}$ |
| **56** | 11 | $K_{11}$ |
| **57** | 35 | $k_{35}$ |
| **58** | 43 | $k_{43}$ |
| **59** | 5 | $K_{5}$ |
| **60** | 5 | $V_{5}$ |
| **61** | 47 | $k_{47}$ |
| **62** | 44 | $k_{44}$ |
| **63** | 4 | $k_{4}$ |
| **64** | 46 | $k_{46}$ |
| **65** | 48 | $k_{48}$ |
| **66** | 43 | $k_{-43}$ |
| **67** | 45 | $k_{-45}$ |
| **68** | 45 | $k_{45}$ |
| **69** | 14 | $K_{14}$ |
| **70** | 31 | $R_{31}$ |
| **71** | 2 | $V_{2}$ |
| **72** | 9 | $V_{9}$ |
| **73** | 27 | $k_{-27}$ |
| **74** | 10 | $K_{10}$ |
| **75** | Non-zero Initial Condition | $[Elk-1]_{0}$ |
| **76** | 29 | $k_{-29}$ |
| **77** | 27 | $k_{27}$ |
